# MiR-20a-5p promotes radio-resistance by targeting NPAS2 in nasopharyngeal cancer cells

**DOI:** 10.18632/oncotarget.22411

**Published:** 2017-11-11

**Authors:** Fangfang Zhao, Youguang Pu, Liting Qian, Chunbao Zang, Zhenchao Tao, Jin Gao

**Affiliations:** ^1^ The Institute of Cancer Research, Anhui Cancer Hospital, West Branch of Anhui Provincial Hospital, Anhui Medical University, Hefei 230031, Anhui, China; ^2^ Department of Radiation Oncology, Anhui Provincial Hospital, Anhui Medical University, Hefei 230031, Anhui, China; ^3^ Department of Radiation Oncology, Anhui Cancer Hospital, West Branch of Anhui Provincial Hospital, Anhui Medical University, Hefei 230031, Anhui, China

**Keywords:** nasopharyngeal cancer, miR-20a-5p, NPAS2, radio-resistance

## Abstract

MicroRNAs (miRNAs) are key players of gene expression involved in diverse biological processes including the cancer radio-resistance, which hinders the effective cancer therapy. Here we found that the miR-20a-5p level is significantly up-regulated in radio-resistant nasopharyngeal cancer (NPC) cells via an RNA-seq and miR-omic analysis. Moreover, we identified that the neuronal PAS domain protein 2 (NPAS2) gene is one of the targets of miR-20a-5p. The involvement of miR-20a-5p and NPAS2 with NPC radio-resistance was further validated by either down- or up-regulation of their levels in NPC cell lines. Taken together, these results not only reveal novel insights into the NPC radio-resistance, but also provide hints for an effective therapeutic strategy to fight against NPC radio-resistance.

## INTRODUCTION

The nasopharyngeal carcinoma (NPC) is a term for a group of malignant tumors that usually happens at the nasopharynx [1]. The current chemo- and radiation therapeutic approaches for NPC are less efficient due to the high sensitivity of NPC [2]. Despite extensive studies on the cancer radio-resistance, the molecular mechanism for NPC radio-resistance remains largely unknown. The failure in radiation treatment mainly results from the production of intrinsic or therapy-triggered resistant tumor cells [3]. Nevertheless, radio-treatment is still commonly used in cancer treatment as it provides increased survival rates due to the excellent local control [4–6]. To conquer the radio-resistance of tumor cells, it is urgently needed to find the key players involved in radio-resistance and develop novel therapeutic strategies.

MicroRNAs (miRNAs) are termed as small non-coding RNA molecules that participate in a wide range of biological events [7]. Several reports have suggested that their dysregulation is associated with the development of many diseases, including cancer of every aspect [8–11]. To date, substantial efforts have been exerted in elucidating the roles of miRNAs in the occurrence and development of radio-resistance in a variety of cancers. For instance, MiR-32 was reported to induce radio-resistance by targeting the DAB2IP gene and regulate autophagy in prostate cancer [12]. MiR-96 was found to promote chemo- or radioresistance by repressing the expression of RECK in esophageal cancer [13]. Moreover, miR-205 sensitized the tumor radio-resistance by targeting Ubc13 and ZEB1 [14]. As one of the well-studied miRNAs, miR-20a belongs to the miR-17-92 cluster, and has been demonstrated to act as an oncomiR in various human cancers, including lung cancer [15], hepatocellular carcinoma [16], as well as gastric cancer [17]. In addition, miR-20, Rest and Wnt signaling are involved in modulating the neural differention of neural progenitor cells [18]. Notably, the expression of miR-20a is also related to irradiation therapy [19]. For example, the higher level of miR-20a activated the PTEN/PI3K/Akt signaling pathway and thus induced radio-resistance of hepatocellular carcinoma [20].

In the present study, to explore the roles of miR-20a-5p in NPC radio-resistance, we performed a RNA-seq based omic analysis to detect the genes that were differentially expressed in radio-sensitive (CNE-2) versus radio-resistant (CNE-1) NPC cell lines. We showed here that miR-20a-5p repressed the NPAS2 gene and promoted NPC radio-resistance. Furthermore, we performed a systematic analysis of miR-20a-5p and NPAS2 for its role in the radio-resistance of NPC cells.

## RESULTS

### The NPAS2 level negatively correlates with the expression of miR-20a-5p in NPC cells

**Figure 1:**
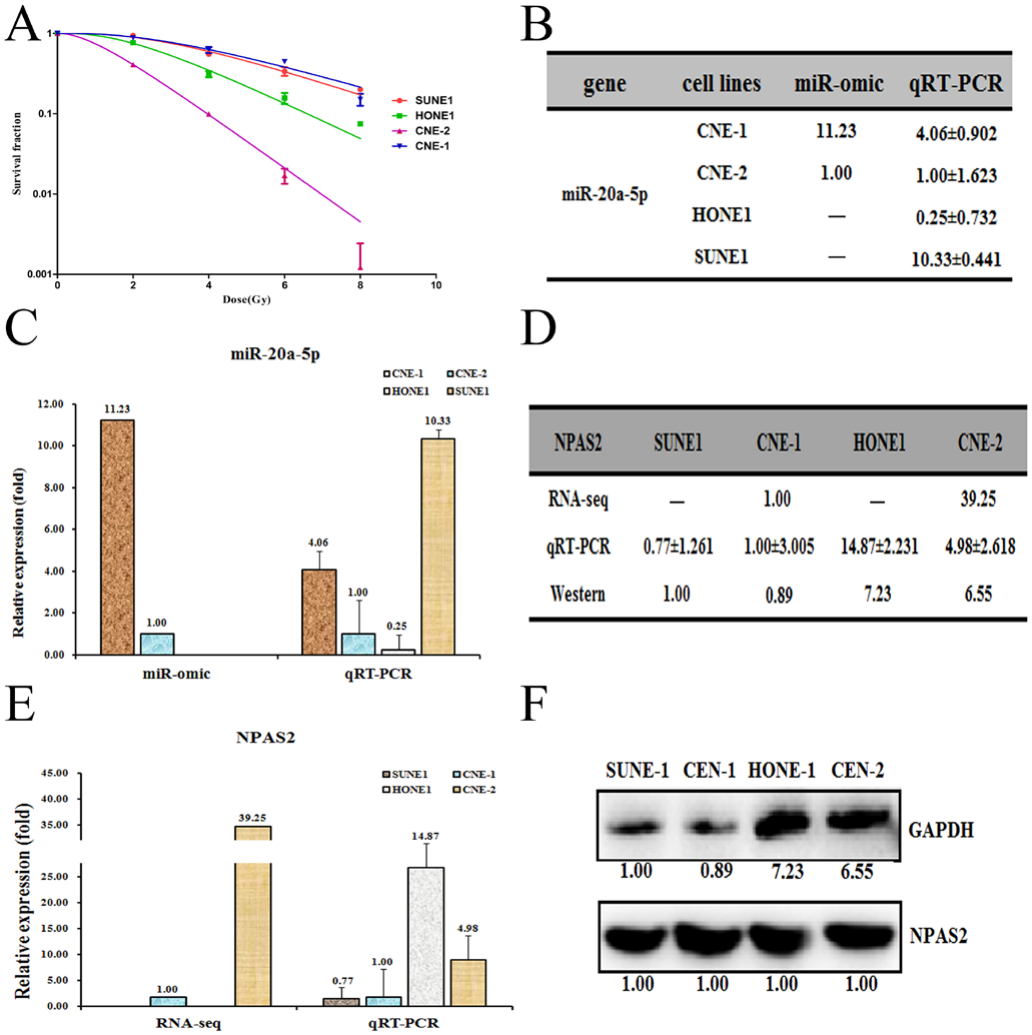
Different expression patterns of miR-20a-5p/NPAS2 in four nasopharyngeal cells SUNE1, HONE1, CNE-1 and CNE-2 **(A)**. The survival fraction of four NPC cells treated as described. The surviving fraction was calculated using the multitarget single-hit model: Y=1-(1-exp(−k^*^x))^N. The data are presented as the mean±standard deviation of results from 3 independent experiments, and two way anova was used to calculate statistical significance. The miR-20a-5p expression levels in four cells (summarized in table **B**) were analyzed by miR-seq and qRT-PCR analyses in plot **(C)**. The expression level of NPAS2 is higher in CNE-2/HONE1 cells than in CNE-1/SUNE1 cells, as summarized in table **(D)**. qRT-PCR and Western blot analyses are shown in plots **(E** and **F)**, respectively.

Previous report demonstrated that CNE-1 and CNE-2 cell lines are the radio-resistant and sensitive cell lines of NPC, respectively [21–23]. Indeed, our radiation treatment experiments against four NPC cells (SUNE1, HONE1, CNE-2 and CNE-1) demonstrated that CNE-1 is the most radio-resistant cells whereas CNE-2 is the most radio-sensitive cell lines (Figure 1A). To reveal the insights that mediate the radio-resistance of NPC, we performed an RNA-seq analysis of CNE-1 and CNE-2 cells. The results showed that several miRNAs were differentially expressed, which were further subject to testing the expression level by qRT-PCR. We selected MiR-20a-5p as our target, which is in agreement with the RNA-seq analyses (Supplementary File 1). The expression of miR-20a-5p is 11.23-fold higher in CNE-1 cells than that in CNE-2 cells by miR-omic analysis. Similar case was also found for the miR-20a-5p level by qRT-PCR analysis (Figure 1B and 1C).

To find the target genes of miR-20a-5p, we next made a prediction using the following websites: TargetScan (http://www.targetscan.org/) and miRDB (http://miRdb.org/miRDB/). We then compared the expression profiles of shared predicted mRNAs between CNE-1 and CNE-2 cells by the RNA-seq based miR-omic analysis. The results showed that a group of genes were drastically differentially expressed in the two cell lines (Supplementary File 1). Among them, the NPAS2 is one of the genes that negatively correlate with the level of miR-20a-5p. We thus selected NPAS2 as our target and further detected the level of NPAS2 in CNE-2 and CNE-1 cells. The results showed that NPAS2 was higher in CNE-2 than CNE-1 at both mRNA (RNA-seq based miR-omic: 39.25:1.00, and qRT-PCR analysis: 4.98:1.00) and protein levels (western blot: 6.55:0.89) (Figure 1D, and 1F).

### MiR-20a-5p targets the NPAS2 gene in NPC cells

**Figure 2:**
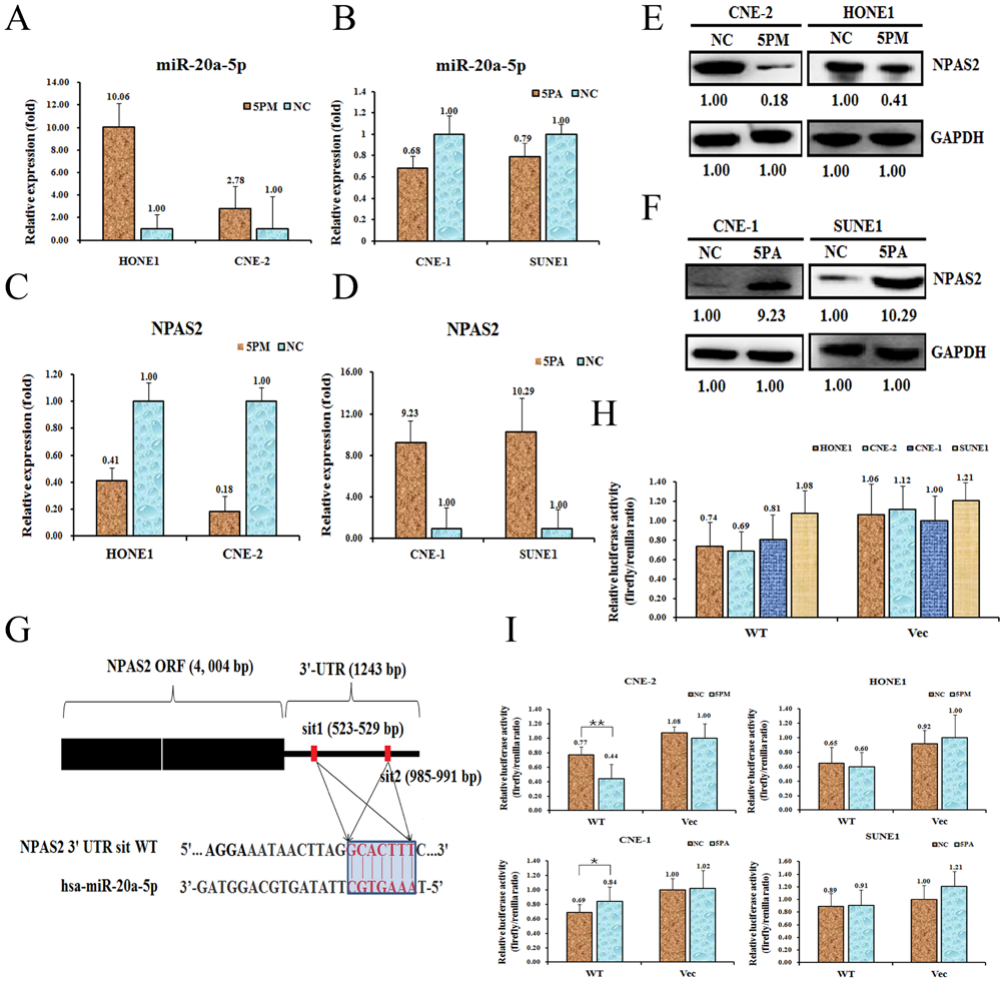
NPAS2 is a target of miR-20a-5p in NPC cells Level of miR-20a-5p **(A** and **B)**. NPAS2 mRNA **(C** and **D)** and protein **(E** and **F)** levels in the miR-20a-5p mimic (5PM)-transfected CNE-2 and HONE1 cells and the miR-20a-5p antagomiR (5PA)-transfected CNE-1 and SONE1 cells versus the negative control (NC) cells, as determined by qRT-PCR or Western blot analyses. Sequences in the UTR region of the NPAS2 gene targeted by miR-20a-5p, with the hatched section showing the combined area **(G)**. The relative luciferase activities (fold) of the reporter with the wild-type (WT) NPAS2-UTR (Vec) were determined in the NPC cells transfected with the miR-20a-5p mimic (in CNE-2 and HONE1), antagomiR (in CNE-1 and SONE1) or Mock **(H** and **I)** sequences. The Renilla luciferase activity of a co-transfected control plasmid was used as a control for the transfection efficiency. The representative results from three independent experiments are shown. ^*^p value<0.05 by Student's *t*-test.

We found that the NPAS2 gene negatively correlates with the level of miR-20a-5p, indicating it might be the target of miR-20a-5p. To test whether NPAS2 is indeed one of the targets of miR-20a-5p, we first determined the NPAS2 level in the miR-20a-5p mimic transfected CNE-2 and HONE1, and the antagomiR transfected CNE-1 and SUNE1 cells versus the NC (scramble sequence control) transfected. Indeed, the transfection of miR-20a-5p mimic into CNE-2 and HONE1 cells increased the level of miR-20a-5p to about 2.78-fold and 10.06-fold, respectively (Figure 2A), whereas the transfection of miR-20a-5p antagomiR into CNE-1 and SUNE1 significantly decreased the expression of miR-20a-5p to about 68% and 79%, respectively (Figure 2B). As a result, the transfection of a miR-20a-5p mimic down-regulated the NPAS2 mRNA to 18% for CNE-2 cells and 41% for HONE1 cells (Figure 2C). As expected, the transfection of miR-20a-5p antagomiR increased the mRNA level of NPAS2 by 9.23 folds for CNE-1 and 10.29 folds for SUNE1 (Figure 2D) Accordingly, the protein levels were also changed at different ratios, which is in agreement with the changes of NPAS2 mRNA levels (Figure 2E, 2F). Sequence analysis found that 3′-UTR region of NPAS2 contains two putative binding motifs for miR-20a-5p (termed sit 1 and sit 2, respectively) (Figure 2G). To further confirm that NPAS2 is a target of miR-20a-5p, we performed a reporter assay by cloning the wild-type NPAS2 gene at the downstream of the Renilla luciferase gene to create the vector of pGL3-NPAS2 UTR WT (Figure 2G). The constructs pGL3-NPAS2 UTR WT was transfected into the four NPC cell lines respectively, to detect the miR-20a-5p-regulated function *in vivo*. The pGL3-NPAS2-UTR WT gave the relative luciferase activities of 0.74, 0.69, 0.81 and 1.08 in HONE1, CNE-2, CNE-1 and SUNE1 cells, respectively (Figure 2H). The transfection of miR-20a-5p-mimic into CNE-2 cells significantly decreased the luciferase activity of pGL3-NPAS2-UTR WT construct, whereas the control cells showed comparable activity (Figure 2I). Meanwhile, the luciferase activity of pGL3-NPAS2-UTR WT construct was higher upon the transfection of miR-20a-5p-antagomiR into CNE-1 cells (Figure 2I). By contrast, the luciferase activities showed slight difference upon the transfection of miR-20a-5p-mimic into HONE1 or miR-20a-5p-antagomiR into SUNE1, as compared to the control cells, probably due to the minor contribution of miR-20a-5p on radio-resistance in these two cell lines. Taken together, NPAS2 is indeed, a target of miR-20a-5p and may execute the miR-20a-5p's promoting effect on the NPC radio-resistance.

### NPAS2 and miR-20a-5p have a reverse effect on NPC radio-resistance

**Figure 3:**
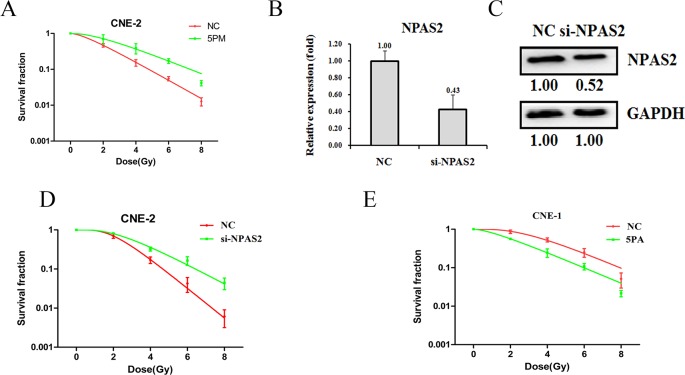
Effects of a forced reversal of the miR-20a-5p or NPAS2 levels on the radio-resistance of CNE-1 and CNE-2 cells MiR-20a-5p mimic (5PM)-transfected CNE-2 increases NC cells survival fraction versus the negative control (NC) cells **(A)**. NPAS2 protein level (Western blot analysis) and mRNA determined by qRT-PCR in the si-NPAS2-transfected versus the NC-transfected CNE-2 cells treated with a 4-Gy dose of radiation **(B** and **C)**. Si-NPAS2-transfected CNE-2 increases NC cells survival fraction versus the negative control (NC) cells **(D)**. MiR-20a-5p antagomiR (5PA)-transfected CNE-1 decreases NC cells survival fraction versus the negative control (NC) cells **(E)**. The surviving fraction was calculated using the multitarget single-hit model: Y=1-(1-exp(−k^*^x))^N. The data are presented as the mean±standard deviation of results from 3 independent experiments, and two way anova was used to calculate statistical significance.

We find that NPAS2 is a target of miR-20a-5p. To further elucidate the roles of miR-20a-5p and NPAS2 on NPC radio-resistance, we performed the cell survival experiments upon the transfection of either miR-20a-5p-mimic or si-NPAS2 into the CNE-2 cells. Accompanied by the increase of miR-20a-5p upon the transfection of miR-20a-5p-mimic in the CNE-2 cells, the cell survival rate was elevated against the radiation treatment (Figure 3A). As expected, the transfection of si-NPAS2 into CNE-2 cells decreased the level of NPAS2 in both mRNA (0.43:1) and protein levels (0.52:1), as compared to the control cells (Figure 3B and 3C). The following results showed an enhanced radio-resistance capability upon the transfection of si-NPAS2, which is reversely correlated with the effect of miR-20a-5p (Figure 3D). On the other hand, the transfection of miR-20a-5p antagomiR in CNE-1 cells decreased the level of miR-20a-5p, which further results in a lower cell survival rate against radiation treatment (Figure 3E). The results clearly demonstrated that NPAS2 negatively regulates NPC radio-resistance, whereas miR-20a-5p has a positive effect on NPC radio-resistance.

**Figure 4:**
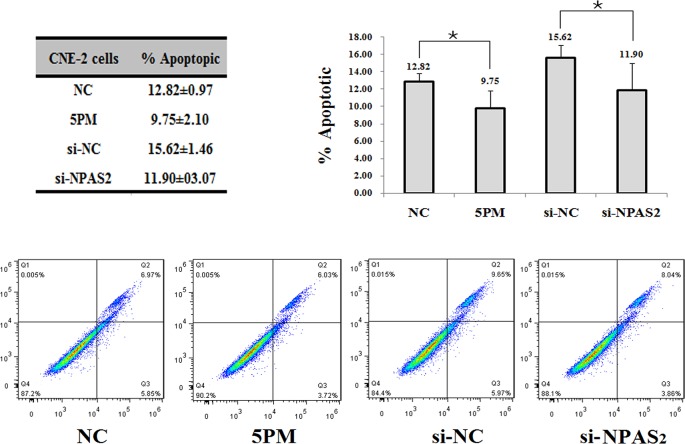
Effects of the forced reversal of both the miR-20a-5p and NPAS2 levels on the apoptosis of CNE-2 cells, with a graph of the analyzed data and plots of the original FACS data (A) ^*^p value < 0.05.

In agreement with its negative effect of NPAS2 on NPC radio-resistance, a siRNA-mediated NPAS2 repression reduced the cell apoptosis rate from 15.62% to 11.90%, which suggests that cell survival rate was elevated upon the addition of si-NPAS2 into CNE-2 cells (Figure 4). Similarly, the miR-20a-5p-mimic transfected CNE-2 cells also showed a reduced cell apotosis rate (Figure 4).

### MiR-20a-5p-mediated NPC radio resistance is probably via the regulation of the Notch signaling pathway

**Figure 5:**
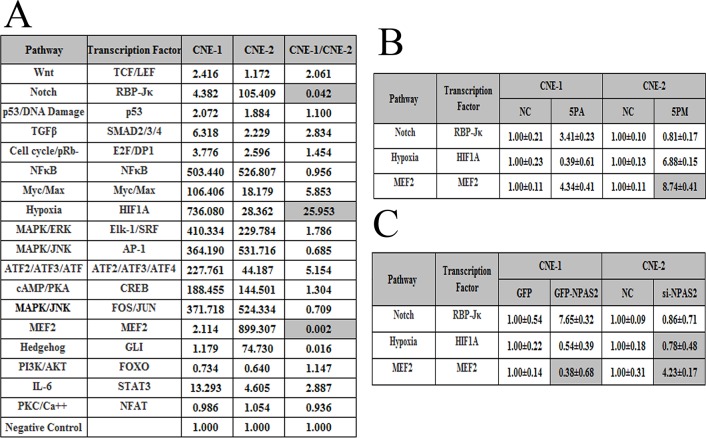
The signaling pathways regulated by miR-20a-5p and their downstream genes The relative activities (mean ± S.D.) of three pathways that differed by more than ten-fold between CNE-1 and CNE-2 cells **(A)**. The relative activities of the pathways in the miR-20a-5p mimic (5PM)-transfected versus NC-transfected CNE-2 cells as well as miR-2a-5p antagomiR (5PA)-transfected versus NC-transfected CNE-1 cells **(B)**. The relative activities of the pathways in the si-NPAS2-transfected versus NC-transfected CNE-2 cells and GFP-NPAS2-transfected versus NC-transfected CNE-1 cells **(C)**.

To further elucidate the molecular mechanisms of NPC radio-resistance, we measured the activities of 18 cancer-related signaling pathways in both CNE-1 and CNE-2 cells (Figure 5A). The pathways with the activities that differed by more than ten folds in CNE-1 and CNE-2 cells were selected for further studies. Among them, the Notch and MEF2 pathways showed higher activities in CNE-2 cells than that in CNE-1 cells, whereas the Hypoxia pathway demonstrated higher activity in CNE-1 cells (Figure 5A). These three most differentially regulated pathways might play a role in NPC radio-resistance. Therefore, we then compared the activities of the three pathways in mimic-transfected CNE-2 cells or antagomiR-transfected CNE-1 cells. The activity of the Hypoxia pathway was elevated in the mimic-transfected CNE-2 cells, whereas it was reduced in the antagomiR-transfected CNE-1 cells (Figure 5B). By contrast, the activity of the Notch pathway was reduced in the miR-20a-5p mimic-transfected CNE-2 cells but increased in the miR-20a-5p antagomiR-transfected CNE-1 cells (Figure 5B). We further compared the pathway activities in si-NPAS2 transfected CNE-2 cells and the GFP-NPAS2 overexpressed CNE-1 cells. The activity of the Notch pathway is elevated upon the transfection of GFP-NPAS2 into CNE-1 cells, whereas decreased upon the transfection of si-NPAS2 into CNE-2 cells (Figure 5C). Thus only the Notch pathway correlates well with the changes of both miR-20a-5p and NPAS2, indicating its involvement with NPC radio-resistance. All together, the Notch pathway was proposed to be involved in the NPC radio-resistance mediated by miR-20a-5p and NPAS2.

## DISCUSSION

Previous studies have demonstrated that miRNAs are closely related with tumor radio-sensitivity, such as miR-23b, miR-124. MiR-23b regulates autophagy and is also involved in radioresistance of pancreatic cancer [24], MiR-124 targets PRRX1 and sensitizes the radio-therapy of human colorectal cancer cells [25]. Of note, a previou study in hepatocellular carcinoma also found that miR-20a activates the PTEN/PI3K/Akt signaling pathway to induce the cell radio-resistance [20]. Considering the important roles of miRNAs to regulate diverse oncogenic processes, including radio-resistance, it is a promising avenue for furthering our understanding of radiation resistance. Therefore, any attempts to improve our understanding of the molecular mechanisms underlying radiation sensitivity and resistance remain an important direction. In the present study, we showed that miR-20a-5p also participates in the NPC radio-resistance. In addition, we performed a comparative RNA-seq omic analysis of CNE-1 and CNE-2 cell lines and identified that the NPAS2 gene exhibits a negative correlation with the radio-resistance (Figure 3). The roles of miR-20a-5p and NPAS2 in the context of NPC radio-resistance were systematically analysed in cultured cells.

Neuronal PAS domain protein 2 (NPAS2), the largest human core circadian gene, encodes for a transcription factor belonging to the basic helix-loop-helix PAS class [26]. NPAS2 was reported to regulate diverse biological processes that are involved in 24-hr circadian rhythm [27]. Down-regulation of NPAS2 has been demonstrated to disrupt the circadian system, such as the sleep pattern [27, 28]. In addition, previous evidences have suggested that NPAS2 is a tumor suppressor that regulates different pathways, such as apoptosis, cell cycle determination, and DNA damage [29–31]. For example, NPAS2 suppress the transcription of the oncogene c-Myc by regulating the circadian-related gene PER [32]. NPAS2 has also been found to be a prognostic biomarker in breast cancer [31], colorectal cancer [33] and non-Hodgkin's lymphoma [34]. All these reports suggest that the inhibition of NPAS2 serves as a therapeutic target for cancers. In agreement with previous findings, here we showed that the NPAS2 level is correlated with the radio-resistance of NPC cells, which might be mediated by miR-20a-5p. However, the fine mechanism for the miR-20a-5p-regulated NPC radio-resistance remains to be clarified.

Moreover, here we also found that the Notch signaling pathway might be involved in the NPC radio-resistance mediated by miR-20a-5p. The Notch signaling not only plays a role in the determination of cell fate [35], but also involves in maintaining the balance of progenitor/stem cell population, as well as cell proliferation, differentiation and apoptosis [36]. Thus Notch signaling pathway may be involved in carcinogenesis, such as lung cancer [37], colorectal cancer [38], liver cancer [39], breast cancer [40] and etc. In accordance with previous findings, our results suggest that the Notch signaling also involves in NPC radio-resistance, which indicates a multi-functional roles of the Notch signaling pathway.

## MATERIALS AND METHODS

### Cell lines

SUNE1, HONE1, CNE-2 and CNE-1 were supplied by the Cancer Center of Sun Yat-sen University [21]. The four cell lines were cultured in Dulbecco's modified Eagle's medium (Invitrogen, Carlsbad, CA, USA) supplemented with 10% fetal bovine serum (Invitrogen) and 1% glutamine at 37°C in 5% CO_2_.

### RNA-Seq analysis

RNA-seq analysis was performed by BGI-Tech of China, and RNA-seq library preparation and sequencing were performed by BGI (Shenzhen, China). RNA was purified and then was fragmented using divalent cations at an elevated temperature. The first-strand cDNA was synthesized using random hexamer primers and Superscript TMIII (Invitrogen™, Carlsbad, CA, USA). Second-strand cDNA was synthesized in the buffer containing dNTPs, RNaseH, and DNA polymerase I. Short fragments were purified with a QiaQuick PCR extraction kit (Qiagen) and resolved with EB buffer for end reparation and poly(A) addition. The short fragments were subsequently connected using sequencing adapters. After agarose gel electrophoresis, suitable fragments were used as templates for PCR amplification. During the QC steps, an Agilent 2100 Bioanaylzer and an ABI StepOnePlus Real-Time PCR System were used in quantification and qualification of the sample library. Finally, the library (200-bp insert) was sequenced using Illumina HiSeq2000 (Illumina Inc., San Diego, CA, USA). The single-end library was prepared following the protocol of the IlluminaTruSeq RNA Sample Preparation Kit (Illumina) [41].

### Irradiation and clonogenic assay

Cells treated as described were seeded on 6-well plates in triplicate and exposed to radiation at the doses indicated using a 6-MV x-ray generated by a linear accelerator (Varian trilogy at a dose rate of 200 cGy/min). After incubation at 37°C for 14 days, cells were fixed in 100% methanol and stained with 0.1% crystal violet. Colonies containing >50 cells were counted under a light microscope. The surviving fraction was calculated as described previously [13, 20]. At least three independent experiments were performed.

### Reagents for the transient transfection assays

The *Homo sapien* (hsa)-miR-20a-5p mimic, antagomiR and scrambled negative control (NC) were supplied by Guangzhou Ribobio, China. MiR-20a-5p mimics and antagomiRs were used to increase and decrease the expression of miR-20a-5p, respectively. miRNA transfection was performed using the riboFECT CP transfection kit were supplied by Guangzhou Ribobio, China. In brief, cells were plated in a 6-well plate and incubated overnight to achieve about 70% confluence at the time of transfection. In each well, 2.5 μl miRNA (100 μM) was added to 120 μl buffer, then 12 μl riboFECT CP transfection reagent was added and mixed gently. The transfection complex was added to the cells and incubated for 48 hr at 37°C in a 5% CO_2_ incubator. To confirm the effect of the miRNAs on the expression of miR-20a-5p, western and RT-qPCR were performed. The sequences used in this study are as follows:

si-NPAS2:

5′-GAAUCAAGUGUGAUAUCAA dTdT-3′

3′-dTdT CUUAGUUCACACUAUAGUU-5′

hsa-miR-20a-5p

antagomiR: 5′-CUACCUGCACUAUAAGCACUUUA-3′

mimic:

sense 5′-UAAAGUGCUUAUAGUGCAGGUAG-3′

antisense 5′-CUACCUGCACUAUAAGCACUUUA-3′.

### RNA analysis

Total RNA was extracted using TRIzol (Invitrogen), according to the manufacturer's instructions. For the mRNA analysis, the cDNA primed by oligo-dT was made with a prime Script RT reagent kit (Tiangen Biotech Co., Ltd., Beijing, China), and the mRNA level of NPAS2 was quantified by a duplex-qRT-PCR analysis where the TaqMan probes with a different fluorescence for β-actin (provided by Shing Gene, Shanghai, China) were used in the FTC-3000P PCR instrument (Funglyn Biotech Inc., Canada). The miRNA expression level was normalized using U6 small nuclear RNA (HmiRQP9001) as an internal control, as previously described [42]. Using the 2−^ΔΔ^Ct method, the normalization with the β-actin level was performed before the relative level of the target genes was compared. The sequences of primers and probes used for the qRT-PCR analysis are as follows: hNPAS2 F: 5′- AGCCCGAGTTCATCGTGTG −3′ hNPAS2 R: 5′- CTTGAGCCCTTGTCCTTTAGTG −3′ hNPAS2 probe: 5′-ROX- CTCGGTGGTCAGTTACGCAGATGTCC −3′ hACTB F: 5′-GCCCATCTACGAGGGGTATG-3′ hACTB R: 5′-GAGGTAGTCAGTCAGGTCCCG-3′ hACTB probe: 5′-CY5-CCCCCATGCCATCCTGCGTC-3′.

### Western blotting assays

Proteins were separated by electrophoresis based on its molecular weight and transferred from the gel to a PVDF membrane.Anti-NPAS2 (YT5045) was purchased from ImmunoWay. The target proteins were then probed with anti-rabbit IgG peroxidase-conjugated antibody (SA00001-2; San Ying Biotechnology, China). The target bands were revealed by an enhanced chemiluminescence reaction (Pierce), and the relative density (level) of proteins over the GAPDH (10494-1-AP; San Ying Biotechnology, China) band was quantified with the Gel-Pro Analyzer (Media Cybernetics).

### Apoptosis analysis

Apoptosis was analyzed by flow cytometry using Annexin V/PI double staining. 48 hr after transfection, the cells were harvested and rinsed with PBS twice, then 3 μl of FITC-labeled enhanced annexinV and 3 μl (20 μg/ml) of propidium iodide were added to 150 μl of cell suspension. After incubation in the dark for 30 min at room temperature, flow cytometry was performed on a FACSCalibur instrument. The number of apoptotic and necrotic cells were calculated by flow cytometry (Becton-Dickinson Co, USA) and analyzed by Flowjo Software. The experiments were performed independently three times, and a representative is shown.

### Luciferase reporter assay

A full-length of the human NPAS2 3′-untranslated region (UTR, 1243 bp) with the target sequence for miR-20a-5p was cloned into the 3′ flank of the luciferase coding sequence of pGL3 (Invitrogen) to construct pGL3-luc-NPAS2 UTR WT. Cells were seeded into 96-well plates at approximately 1×10^4^ cells per well and cotransfected with a mixture of 50 ng of pGL3-luc-NPAS2 UTR WT, plus 5 pmol of mimic or NC nucleotides using the riboFECT CP transfection kit according to the manufacturer's instruction. The luciferase activities were measured 24 hr after transfection by the Dual-Luciferase Reporter Assay System (Promega) using a Promega GloMax 20/20 luminometer. The relative firefly luciferase activities of the UTR construct and pathway reporter constructs were analyzed as previously reported [43].

### Signaling pathway analysis

The signaling pathway assays was finished with SABiosciences (USA) reagent kit, which contains the NC construct, the reporter construct and the positive control construct. The analysis was performed according to the manufacturer's instruction. Briefly, the cells were triple transfected with each firefly luciferase reporter construct in combination with the Renilla luciferase construct using the riboFECT CP transfection reagent, and both luciferase activities in cell extracts at 24 hr after transfection were measured using the Promega Dual-Luciferase Reporter assay on the PromegaGloMax 20/20 luminometer. Firefly luciferase activities from each set were normalized to the Renilla luciferase activity to control for inter-transfection bias. The relative luciferase activities (luciferase unit) of the pathway reporter over the negative control in the transfected cells were calculated as a measure of the pathway activity.

### Statistical analyses

The data are presented as the mean, and the error bars indicate the S.D. All statistical analyses were performed with GraphPad Prism 5. Two way anova and two-tailed Student's *t*-test were used to calculate statistical significance. p-value of<0.05 was considered significant.

## SUPPLEMENTARY MATERIALS FIGURE



## References

[1] Liu T (1999). Issues in the management of nasopharyngeal carcinoma. Crit Rev Oncol Hematol.

[2] Horsman MR, Bohm L, Margison GP, Milas L, Rosier JF, Safrany G, Selzer E, Verheij M, Hendry JH (2006). Tumor radiosensitizers--current status of development of various approaches: report of an International Atomic Energy Agency meeting. Int J Radiat Oncol Biol Phys.

[3] Jameel JK, Rao VS, Cawkwell L, Drew PJ (2004). Radioresistance in carcinoma of the breast. Breast.

[4] Louis C, Dewas S, Mirabel X, Lacornerie T, Adenis A, Bonodeau F, Lartigau E (2010). Stereotactic radiotherapy of hepatocellular carcinoma: preliminary results. Technol Cancer Res Treat.

[5] Seo YS, Kim MS, Yoo SY, Cho CK, Choi CW, Kim JH, Han CJ, Park SC, Lee BH, Kim YH, Lee DH (2010). Preliminary result of stereotactic body radiotherapy as a local salvage treatment for inoperable hepatocellular carcinoma. J Surg Oncol.

[6] Tse RV, Hawkins M, Lockwood G, Kim JJ, Cummings B, Knox J, Sherman M, Dawson LA (2008). Phase I study of individualized stereotactic body radiotherapy for hepatocellular carcinoma and intrahepatic cholangiocarcinoma. J Clin Oncol.

[7] Pillai RS, Bhattacharyya SN, Filipowicz W (2007). Repression of protein synthesis by miRNAs: how many mechanisms?. Trends Cell Biol.

[8] Lu J, Getz G, Miska EA, Alvarez-Saavedra E, Lamb J, Peck D, Sweet-Cordero A, Ebert BL, Mak RH, Ferrando AA, Downing JR, Jacks T, Horvitz HR, Golub TR (2005). MicroRNA expression profiles classify human cancers. Nature.

[9] Volinia S, Calin GA, Liu CG, Ambs S, Cimmino A, Petrocca F, Visone R, Iorio M, Roldo C, Ferracin M, Prueitt RL, Yanaihara N, Lanza G (2006). A microRNA expression signature of human solid tumors defines cancer gene targets. Proc Natl Acad Sci U S A.

[10] Niemoeller OM, Niyazi M, Corradini S, Zehentmayr F, Li M, Lauber K, Belka C (2011). MicroRNA expression profiles in human cancer cells after ionizing radiation. Radiat Oncol.

[11] Weidhaas JB, Babar I, Nallur SM, Trang P, Roush S, Boehm M, Gillespie E, Slack FJ (2007). MicroRNAs as potential agents to alter resistance to cytotoxic anticancer therapy. Cancer Res.

[12] Liao H, Xiao Y, Hu Y, Xiao Y, Yin Z, Liu L (2015). microRNA-32 induces radioresistance by targeting DAB2IP and regulating autophagy in prostate cancer cells. Oncol Lett.

[13] Xia H, Chen S, Chen K, Huang H, Ma H (2014). MiR-96 promotes proliferation and chemo- or radioresistance by down-regulating RECK in esophageal cancer. Biomed Pharmacother.

[14] Zhang P, Wang L, Rodriguez-Aguayo C, Yuan Y, Debeb BG, Chen D, Sun Y, You MJ, Liu Y, Dean DC, Woodward WA, Liang H, Yang X (2014). miR-205 acts as a tumour radiosensitizer by targeting ZEB1 and Ubc13. Nat Commun.

[15] Hayashita Y, Osada H, Tatematsu Y, Yamada H, Yanagisawa K, Tomida S, Yatabe Y, Kawahara K, Sekido Y, Takahashi T (2005). A polycistronic microRNA cluster, miR-17-92, is overexpressed in human lung cancers and enhances cell proliferation. Cancer Res.

[16] Kutay H, Bai S, Datta J, Motiwala T, Pogribny I, Frankel W, Jacob ST, Ghoshal K (2006). Downregulation of miR-122 in the rodent and human hepatocellular carcinomas. J Cell Biochem.

[17] Li X, Zhang Z, Yu M, Li L, Du G, Xiao W, Yang H (2013). Involvement of miR-20a in promoting gastric cancer progression by targeting early growth response 2 (EGR2). Int J Mol Sci.

[18] Cui Y, Han J, Xiao Z, Chen T, Wang B, Chen B, Liu S, Han S, Fang Y, Wei J, Wang X, Ma X, Dai J (2016). The miR-20-Rest-Wnt signaling axis regulates neural progenitor cell differentiation. Sci Rep.

[19] Wagner-Ecker M, Schwager C, Wirkner U, Abdollahi A, Huber PE (2010). MicroRNA expression after ionizing radiation in human endothelial cells. Radiat Oncol.

[20] Zhang Y, Zheng L, Ding Y, Li Q, Wang R, Liu T, Sun Q, Yang H, Peng S, Wang W, Chen L (2015). MiR-20a induces cell radioresistance by activating the PTEN/PI3K/Akt signaling pathway in hepatocellular carcinoma. Int J Radiat Oncol Biol Phys.

[21] Li J, Tu Z, Shen Z, Xia Y, He Y, Liu S, Chen C (2013). Quantitative measurement of optical attenuation coefficients of cell lines CNE1, CNE2, and NP69 using optical coherence tomography. Lasers Med Sci.

[22] He BF, Sun AM, Huang BY, Wang WJ, Zheng XK, Luo RC (2011). [Gefitinib enhances the radiosensitivity of nasopharyngeal carcinoma cell line CNE2 *in vitro*]. [Article in Chinese]. Nan Fang Yi Ke Da Xue Xue Bao.

[23] Hui ZG, Li YX, Yang WZ, Wu JX, Yu ZH (2003). [Abrogation of radiation-induced G2 arrest and radiosensitization by 7-hydroxystaurosporine (UCN-01) in human nasopharyngeal carcinoma cell line]. [Article in Chinese]. Ai Zheng.

[24] Wang P, Zhang J, Zhang L, Zhu Z, Fan J, Chen L, Zhuang L, Luo J, Chen H, Liu L, Chen Z, Meng Z (2013). MicroRNA 23b regulates autophagy associated with radioresistance of pancreatic cancer cells. Gastroenterology.

[25] Zhang Y, Zheng L, Huang J, Gao F, Lin X, He L, Li D, Li Z, Ding Y, Chen L (2014). MiR-124 radiosensitizes human colorectal cancer cells by targeting PRRX1. PLoS One.

[26] Zhou YD, Barnard M, Tian H, Li X, Ring HZ, Francke U, Shelton J, Richardson J, Russell DW, McKnight SL (1997). Molecular characterization of two mammalian bHLH-PAS domain proteins selectively expressed in the central nervous system. Proc Natl Acad Sci U S A.

[27] Reick M, Garcia JA, Dudley C, McKnight SL (2001). NPAS2: an analog of clock operative in the mammalian forebrain. Science.

[28] Dudley CA, Erbel-Sieler C, Estill SJ, Reick M, Franken P, Pitts S, McKnight SL (2003). Altered patterns of sleep and behavioral adaptability in NPAS2-deficient mice. Science.

[29] Zmrzljak UP, Rozman D (2012). Circadian regulation of the hepatic endobiotic and xenobitoic detoxification pathways: the time matters. Chem Res Toxicol.

[30] Hoffman AE, Zheng T, Ba Y, Zhu Y (2008). The circadian gene NPAS2, a putative tumor suppressor, is involved in DNA damage response. Mol Cancer Res.

[31] Yi C, Mu L, de la Longrais IA, Sochirca O, Arisio R, Yu H, Hoffman AE, Zhu Y, Katsaro D (2010). The circadian gene NPAS2 is a novel prognostic biomarker for breast cancer. Breast Cancer Res Treat.

[32] Fu L, Pelicano H, Liu J, Huang P, Lee C (2002). The circadian gene Period2 plays an important role in tumor suppression and DNA damage response *in vivo*. Cell.

[33] Xue X, Liu F, Han Y, Li P, Yuan B, Wang X, Chen Y, Kuang Y, Zhi Q, Zhao H (2014). Silencing NPAS2 promotes cell growth and invasion in DLD-1 cells and correlated with poor prognosis of colorectal cancer. Biochem Biophys Res Commun.

[34] Zhu Y, Leaderer D, Guss C, Brown HN, Zhang Y, Boyle P, Stevens RG, Hoffman A, Qin Q, Han X, Zheng T (2007). Ala394Thr polymorphism in the clock gene NPAS2: a circadian modifier for the risk of non-Hodgkin's lymphoma. Int J Cancer.

[35] Artavanis-Tsakonas S, Rand MD, Lake RJ (1999). Notch signaling: cell fate control and signal integration in development. Science.

[36] Schroeter EH, Kisslinger JA, Kopan R (1998). Notch-1 signalling requires ligand-induced proteolytic release of intracellular domain. Nature.

[37] Konishi J, Kawaguchi KS, Vo H, Haruki N, Gonzalez A, Carbone DP, Dang TP (2007). Gamma-secretase inhibitor prevents Notch3 activation and reduces proliferation in human lung cancers. Cancer Res.

[38] Veenendaal LM, Kranenburg O, Smakman N, Klomp A, Borel Rinkes IH, van Diest PJ (2008). Differential Notch and TGFbeta signaling in primary colorectal tumors and their corresponding metastases. Cell Oncol.

[39] Qi R, An H, Yu Y, Zhang M, Liu S, Xu H, Guo Z, Cheng T, Cao X (2003). Notch1 signaling inhibits growth of human hepatocellular carcinoma through induction of cell cycle arrest and apoptosis. Cancer Res.

[40] Reedijk M, Odorcic S, Chang L, Zhang H, Miller N, McCready DR, Lockwood G, Egan SE (2005). High-level coexpression of JAG1 and NOTCH1 is observed in human breast cancer and is associated with poor overall survival. Cancer Res.

[41] Tarazona S, Garcia-Alcalde F, Dopazo J, Ferrer A, Conesa A (2011). Differential expression in RNA-seq: a matter of depth. Genome Res.

[42] Liu K, Huang J, Xie M, Yu Y, Zhu S, Kang R, Cao L, Tang D, Duan X (2014). MIR34A regulates autophagy and apoptosis by targeting HMGB1 in the retinoblastoma cell. Autophagy.

[43] Lv L, Deng H, Li Y, Zhang C, Liu X, Liu Q, Zhang D, Wang L, Pu Y, Zhang H, He Y, Wang Y, Yu Y (2014). The DNA methylation-regulated miR-193a-3p dictates the multi-chemoresistance of bladder cancer via repression of SRSF2/PLAU/HIC2 expression. Cell Death Dis.

