# Supplementary material for: MiR-20a-5p promotes radio-resistance by targeting NPAS2 in nasopharyngeal cancer cells
Source: Oncotarget. 2017 Nov 11;8(62):105873–81. doi: 10.18632/oncotarget.22411 (PMC5739686; doi:10.18632/oncotarget.22411)
Supplement: Supplementary file 1 [file oncotarget-08-105873-s001.pdf]

MiR-20a-5p promotes radio-resistance by targeting NPAS2 in nasopharyngeal cancer cells

SUPPLEMENTARY MATERIALS

A

miR_name	CNE-2	CNE-1	CNE-1/CNE-2
hsa-miR-218-5p	5.00	186.00	37.20
hsa-miR-362-5p	1.00	27.00	27.00
hsa-miR-1294	1.00	16.00	16.00
hsa-miR-215-5p	18.00	261.00	14.50
hsa-miR-20a-5p	4.54	51.00	11.23
hsa-miR-455-3p	2.00	21.00	10.50
hsa-miR-2682-5p	25.00	234.00	9.36
hsa-miR-3660	4.00	37.00	9.25
hsa-miR-3607-3p	1.00	9.00	9.00
hsa-miR-4788	3.00	26.00	8.67
hsa-miR-3690	3.00	26.00	8.67
hsa-miR-4511	7.00	58.00	8.29
hsa-miR-301b-5p	1.00	8.00	8.00
hsa-miR-30c-2-3p	756.00	5797.00	7.67
hsa-miR-3126-5p	4.00	30.00	7.50
hsa-miR-6807-3p	2.00	15.00	7.50
hsa-miR-548f-3p	478.00	3487.00	7.29
hsa-miR-26a-1-3p	4.00	28.00	7.00
hsa-miR-548q	1.00	7.00	7.00
hsa-miR-4737	1.00	7.00	7.00
hsa-miR-5189-5p	1.00	7.00	7.00
hsa-miR-3610	1.00	7.00	7.00
hsa-miR-4687-5p	1.00	7.00	7.00
hsa-miR-139-3p	1.00	7.00	7.00
hsa-miR-6772-3p	3.00	19.00	6.33
hsa-miR-30a-5p	85028.00	529007.00	6.22
hsa-miR-30a-3p	5283.00	32597.00	6.17
hsa-miR-450b-5p	72.00	441.00	6.13
hsa-miR-4645-3p	3.00	18.00	6.00
hsa-miR-95-5p	2.00	12.00	6.00
hsa-miR-4517	1.00	6.00	6.00
hsa-miR-3179	1.00	6.00	6.00
hsa-miR-550a-3-5p	1.00	6.00	6.00
hsa-miR-6728-5p	1.00	6.00	6.00

B

symbol	CNE-1	CNE-2	CNE-2/CNE-1
C10orf114	0.001	1.317	994.340
C6orf70	0.007	3.421	499.720
KLK5	0.107	17.762	165.580
KLK10	0.309	18.207	58.940
KLK11	0.326	17.455	53.570
CES1	0.114	5.080	44.710
GDF6	0.152	6.129	40.360
NPAS2	10.542	413.762	39.250
ROR2	0.099	3.587	36.380
LY6D	4.379	155.202	35.440
PDE3A	0.090	2.235	24.720
CA9	1.013	22.775	22.470
KLHL13	0.041	0.876	21.550
ABP1	0.700	14.884	21.260
NPNT	0.220	4.595	20.910
KRT6A	1.844	37.715	20.450
FXYD3	15.475	249.630	16.130
UGT1A6	5.717	91.011	15.920
KRT13	43.600	689.547	15.820
ABCA12	0.090	1.368	15.210
KRT5	4.498	67.936	15.100
DSC3	0.189	2.604	13.770
MBNL3	0.034	0.470	13.710
KANK4	0.269	3.504	13.020
RGMA	0.052	0.636	12.340
IL20RA	0.263	3.006	11.430
KRT4	1.373	15.677	11.410
COL17A1	15.015	168.536	11.220
VANGL2	0.057	0.641	11.160
LCN2	2.998	33.390	11.140
SYK	0.619	6.890	11.130
KLK6	1.137	12.642	11.120

Supplementary File 1: The interested miRNA and mRNA genes based on the websites and RNA-seq analysis. A dozen of miRNAs were differentially expressed in the radio-resistant NPC cells CNE-1 and the radio-sensitive NPC cells CNE-2 based on the websites, and the ratio over 6 of CNE-1/CNE-2 based on miR-omic analysis were showed in descending order, has-miR-20a-5p was one of them **(A)**. Reference to similar methods, the downstream genes of miR-20a-5p were also showed, the ratio over 11 of CNE-2/CNE-1 based on RNA-seq analysis were showed in descending order, NPAS2 is located **(B)**.
